# Performance of risk prediction for inflammatory bowel disease based on genotyping platform and genomic risk score method

**DOI:** 10.1186/s12881-017-0451-2

**Published:** 2017-08-29

**Authors:** Guo-Bo Chen, Sang Hong Lee, Grant W. Montgomery, Naomi R. Wray, Peter M. Visscher, Richard B. Gearry, Ian C. Lawrance, Jane M. Andrews, Peter Bampton, Gillian Mahy, Sally Bell, Alissa Walsh, Susan Connor, Miles Sparrow, Lisa M. Bowdler, Lisa A. Simms, Krupa Krishnaprasad, the International IBD Genetics Consortium, Graham L. Radford-Smith, Gerhard Moser

**Affiliations:** 1 Queensland Brain Institute, The University of Queensland, Brisbane, Australia; 2 School of Environmental and Rural Science, The University of New England, Armidale, Australia; 3 Institute for Molecular Bioscience, The University of Queensland, Brisbane, Australia; 4 University of Queensland Diamantina Institute, Translational Research Institute, The University of Queensland, Brisbane, Australia; 5 Department of Medicine, University of Otago, Christchurch, New Zealand; 6 Department of Gastroenterology, Christchurch Hospital, Christchurch, New Zealand; 7 Harry Perkins Institute of Medical Research, School of Medicine and Pharmacology, University of Western Australia, Murdoch, Australia; 8 Centre for Inflammatory Bowel Diseases, Saint John of God Hospital, Subiaco, Australia; 9 Inflammatory Bowel Disease Service, Department of Gastroenterology and Hepatology, Royal Adelaide Hospital, School of Medicine, University of Adelaide, Adelaide, Australia; 10 Department of Gastroenterology and Hepatology, Flinders Medical Centre, Adelaide, Australia; 11 Department of Gastroenterology, Townsville Hospital, Townsville, Australia; 12 Department of Gastroenterology, St Vincent’s Hospital, Melbourne, Australia; 13 Department of Gastroenterology and Hepatology, St Vincent’s Hospital, Sydney, Australia; 14 Department of Gastroenterology and Hepatology, Liverpool Hospital, Sydney, Australia; 15 University of NSW, Sydney, Australia; 16 Department of Gastroenterology, Alfred Health, Melbourne, Australia; 17 School of Medicine, The University of Queensland, Brisbane, Australia; 18 Inflammatory Bowel Disease Research Group, Immunology Division, QIMR Berghofer Medical Research Institute, Brisbane, Australia; 19 Department of Gastroenterology, Royal Brisbane and Women’s Hospital, Brisbane, Australia

**Keywords:** Inflammatory bowel disease, Crohn’s disease, Ulcerative colitis, Case-control study, Risk score, SNP array, Complex trait

## Abstract

**Background:**

Predicting risk of disease from genotypes is being increasingly proposed for a variety of diagnostic and prognostic purposes. Genome-wide association studies (GWAS) have identified a large number of genome-wide significant susceptibility loci for Crohn’s disease (CD) and ulcerative colitis (UC), two subtypes of inflammatory bowel disease (IBD). Recent studies have demonstrated that including only loci that are significantly associated with disease in the prediction model has low predictive power and that power can substantially be improved using a polygenic approach.

**Methods:**

We performed a comprehensive analysis of risk prediction models using large case-control cohorts genotyped for 909,763 GWAS SNPs or 123,437 SNPs on the custom designed Immunochip using four prediction methods (polygenic score, best linear genomic prediction, elastic-net regularization and a Bayesian mixture model). We used the area under the curve (AUC) to assess prediction performance for discovery populations with different sample sizes and number of SNPs within cross-validation.

**Results:**

On average, the Bayesian mixture approach had the best prediction performance. Using cross-validation we found little differences in prediction performance between GWAS and Immunochip, despite the GWAS array providing a 10 times larger effective genome-wide coverage. The prediction performance using Immunochip is largely due to the power of the initial GWAS for its marker selection and its low cost that enabled larger sample sizes. The predictive ability of the genomic risk score based on Immunochip was replicated in external data, with AUC of 0.75 for CD and 0.70 for UC. CD patients with higher risk scores demonstrated clinical characteristics typically associated with a more severe disease course including ileal location and earlier age at diagnosis.

**Conclusions:**

Our analyses demonstrate that the power of genomic risk prediction for IBD is mainly due to strongly associated SNPs with considerable effect sizes. Additional SNPs that are only tagged by high-density GWAS arrays and low or rare-variants over-represented in the high-density region on the Immunochip contribute little to prediction accuracy. Although a quantitative assessment of IBD risk for an individual is not currently possible, we show sufficient power of genomic risk scores to stratify IBD risk among individuals at diagnosis.

**Electronic supplementary material:**

The online version of this article (doi:10.1186/s12881-017-0451-2) contains supplementary material, which is available to authorized users.

## Background

Inflammatory bowel disease (IBD) is a global disease with the prevalence and incidence for Crohn’s disease (CD) and ulcerative colitis (UC) rapidly increasing worldwide [1]. Some individuals are more predisposed to IBD than others, and genomic testing is appealing for individualised monitoring and disease management. At present, the low prevalence of CD and UC makes it difficult to identify ‘at risk’ individuals.

There are now over 200 loci for CD and UC, identified in GWAS and Immunochip studies using more than 95,000 samples [2, 3]. However, these genome-wide significant loci only account for a modest proportion of the total variation of the diseases. The variance on the liability explained by the significant loci is ~0.13 and ~0.08 for CD and UC, respectively [2, 3].

As for any complex disease, there are many more SNPs associated with phenotype that have small effect sizes and the inclusion of non-genome-wide significant variants is likely to make a positive contribution to the prediction model [4]. Using genome-wide data, a number of studies have assessed risk prediction of CD and predictive ability of the models, as measured by the area under the ROC curve (AUC), ranging from 0.64 to 0.86 [5–9]. Comparison between these studies is difficult due to differences in prediction method, sample size and genotyping chip.

In this report, we performed genomic risk prediction of CD and UC using four prediction methods that utilise genome-wide SNP data. We further investigated how performance was influenced by the size of the discovery sample and the choice of the genotyping platform. We show that genotype-based risk predictors can achieve a substantial separation of cases from controls. We further demonstrate high discriminant power between the top and bottom 10% of individuals ranked on their risk score in an independent cohort and a relationship between genomic risk predictor and severity of CD.

## Methods

The International Inflammatory Bowel Disease Genetics Consortium (IIBDGC, Additional file 1) provided data on over 68,000 IBD patients and 29,000 healthy controls from 15 cohorts of mainly European descent. Initial GWAS and subsequent meta-analyses used genome-wide SNP arrays and imputed SNPs [10, 11], but the majority of samples were genotyped with Immunochip [2].

### SNP arrays and quality control

We received 1,253,071 and 1,253,093 imputed GWAS SNPs for CD and UC, respectively. For convenience we refer to these genotypes as gChip. After following the quality control (QC) protocol provided by IIBDGC, we performed additional QC steps, retaining SNPs with imputation quality INFO score R^2^ > 0.6 and minor allele frequency (MAF) > 0.01 in each of the imputation cohorts for CD (*N* = 6) and UC (*N* = 7), and identified 987,572 SNPs that were in common between CD and UC samples.

**Fig. 1:**
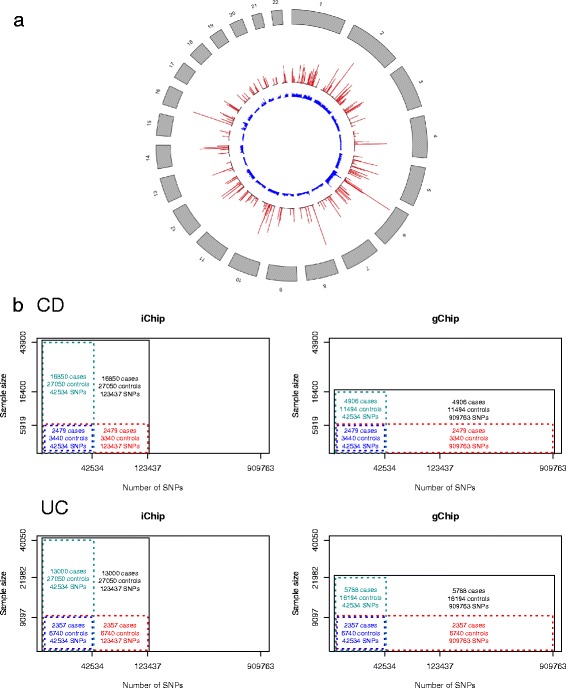
Datasets used in this study. **a** SNP density of iChip and gChip SNPs. The whole genome was partitioned into 0.6 M bins on each chromosome. The middle and inner circles indicate the density of the SNPs on iChip and gChip, respectively. The spikes for iChip depict regions of dense coverage mainly chosen for replication and fine mapping of GWAS loci, while gChip provides a uniform coverage with higher average density. **b** Partitioning of data into sets of increasing sample size and number of SNPs. Samples were split into four subsets with increasing number of individuals and SNPs. The smallest subsets (*dotted box*) include samples genotyped on both gChip and iChip and SNPs overlapping between chips

The data we received for Immunochip (iChip) comprised 176,709 SNPs. Initial quality control followed the preliminary guidelines provided by IIBDGC [2]. In addition we eliminated SNPs with *P-*values <1*e*-6 in the test of Hardy-Weinberg proportions, SNPs with MAF less than 0.001, and individuals with >2% missing genotypes. Due to the low effective number of markers on iChip (Fig. 1a), a relatedness threshold of 0.2 (equivalent to about 0.05 for gChip [12]) was set to remove one member at random from a pair of related individuals.

To rule out potential mistakes in risk prediction, SNPs with palindromic alleles (A/T, G/C) were removed from iChip and gChip. After quality control 123,437 SNPs for iChip and 909,763 SNPs for gChip were available to evaluate predictors for CD and UC. The number of overlapping SNPs between iChip and gChip was 42,534 (42 K set, Fig. 1a). All analyses used only autosomal SNPs.

To summarise the differences between the 42 K SNP set, iChip and gChip, we calculated the effective number of markers (i.e. quasi-linkage equilibrium markers) from the genomic relationship matrix as described in [12]. The number of independent SNPs was 2750 for the 42 K SNP set, 2986 for iChip and 37,226 for gChip.

### Partitioning of case-control samples

The data sets used in this study are described in Fig. 1b. The discovery population for gChip included 16,400 individuals for CD (4906 cases and 11,494 controls) and 21,982 for UC (5788 cases and 16,194 controls). For iChip 16,850 CD cases, 13,000 UC cases, and 27,050 common controls were available as discovery samples. Individuals recruited from Australia and New Zealand (ANZ cohort) were not included in the discovery sample and served as an independent validation dataset. The ANZ cohort consists of 1193 UC and 2204 CD cases and 997 common controls.

For each trait, we considered various scenarios differing in the number of discovery samples, number of SNPs and genotyping platform (Fig. 1b). We first created discovery sets by extracting individuals that were genotyped with both iChip and gChip and limited the marker panel to the 42 K SNPs genotyped on both genomic platforms. These data served as a baseline to assess how performance changed with increasing sample size and increasing SNP coverage.

### Genomic risk prediction methods

We applied four different methods for whole-genome marker-enabled prediction. Genetic profile risk scores (GPRS) were constructed using the effects of all SNPs estimated from single-marker association analyses using PLINK [13]. An alternative to GPRS is a best linear genomic prediction (GBLUP [14]) which is based on mixed linear model that regresses phenotypes on all SNPs jointly. For GBLUP we used the MTG2 software [15, 16]. The third method applied elastic net regularization (EN) using the *glmnet* package [17] in R [18]. The EN method was recently applied by Wei et al. [8] for risk prediction of CD and UC using the IIBDGC iChip data. When applying EN, we first performed a single SNP association analysis using PLINK and then restricted the model space to the 8000 most significant SNPs, followed by 10-fold cross-validation to choose the optimal EN tuning parameter. We also applied BayesR [19, 20], which uses a Bayesian hierarchical method that models SNP effects as a mixture of normal distributions. To be able to fit the BayesR model to the large datasets in this study we developed a more efficient algorithm implemented in a newer version of the BayesR software. Prior assumptions and MCMC parameters for BayesR were as described in [20]. For the case-control data, a generalised linear model with a logit link function was used for GPRS and EN, whereas a linear mixed model was used for GBLUP and BayesR.

We also tried to apply Bayesian Sparse Linear Mixed Models [21] but encountered a run time error (segmentation fault) for the datasets with more than 20,000 individuals using GEMMA v0.94. Another method we investigated was the multiBLUP method developed by Speed and Balding [22], which extends the GBLUP method to several variance components and was reported to increase prediction accuracy of CD in the Wellcome Trust Case Control Consortium dataset [22]. However, using Adaptive multiBLUP implemented in LDAK v4.9, we observed that prediction accuracy was generally lower than GBLUP for the same training sets (Additional file 2: Figure S1). Such behaviour is unexpected as the GBLUB model can be considered the ‘baseline’ model of multiBLUP. We therefore do not report multiBLUP results in the main text.

### Prediction performance

GPRS, EN, GBLUP and BayesR were used to predict risk of CD and UC in each of the different data sets illustrated in Fig. 1b. Prediction performance was assessed by 5-fold cross-validation. Each data set was partitioned in K = 5 folds. In each iteration, 4 of the 5 folds were used as a training set to train a different model for each method, while the 5th fold was used to test the models. This process was repeated 5 times, with a different fold used for testing in each case. Accuracy of risk prediction was measured by averaging the area under the ROC curve (AUC [23]) over the K left-out folds. To ensure that predictive performance was not biased by population structure, we regressed disease phenotype on the top 10 projected eigenvectors estimated from the POPRES reference panel ([24], Additional file 3: Figure S2) and repeated the analysis using the residuals from the adjusted phenotypes. The top eigenvectors of our samples were projected from a sample of 2466 Europeans from the POPRES reference panel using 608,435 SNPs genotyped on gChip. For samples genotyped with iChip we used their projected eigenvectors with gChip SNPs for adjusting phenotypes.

We also evaluated the capability of genomic risk score, which is a continuous score, to predict case-control status in the ANZ cohort which is not part of the discovery population for CD and UC. For this purpose we categorised the scores into deciles and estimated the odds ratio of case-control status by contrasting each decile to the lowest decile. Odds ratio of case-control status was calculated for the largest iChip models, which included 123,437 SNPs and 43,900 and 40,050 individuals for CD and UC, respectively.

Finally, we investigated the association between genomic risk score and known risk stratification factors for CD in a group of 823 patients from the ANZ cohort (Additional file 4: Table S1) by regressing risk score on risk factor, including time of onset (1–19 years, 20–39 years, > 40 years), need of bowel surgery (no, yes) and disease location (ileal only, colon only, ileocolonic).

## Results

We considered various scenarios to assess the utility of genomic risk prediction models for CD and UC depending on genomic risk score method, genotyping platform and size of the discovery sample (Fig. 1).

### Common individuals and common SNPs between iChip and gChip

Our initial analyses were restricted to individuals genotyped on both iChip and gChip (2479 cases and 3440 controls for CD; 2357 cases and 6740 controls for UC) and 42,534 SNPs (42 K) that were in common between platforms. To evaluate risk prediction performance we performed within study 5-fold CV.

**Fig. 2:**
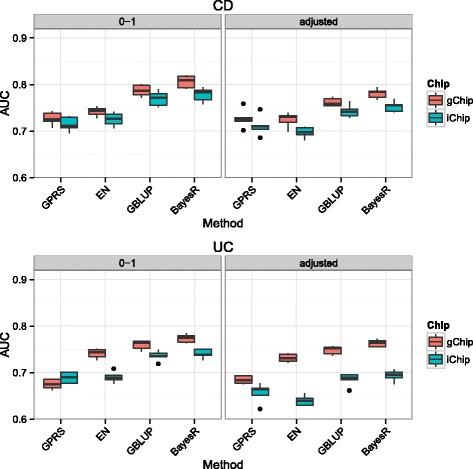
Comparison of prediction performance of four methods using individuals and SNPs common between gChip and iChip. The sample consisted of 2479 cases and 3440 controls for CD and 2357 cases and 6740 controls for UC. The number of SNPs was 42,534. Prediction accuracy is measured as the area under the curve (AUC) with higher values denoting better performance. Vertical lines display the variation of estimates in 5-fold cross-validation. Prediction models were trained using either disease status (0–1) or disease phenotype adjusted for ancestry (adjusted)

We found that BayesR performed better in prediction than alternative methods (Fig. 2). Compared to the GPRS method, using BayesR led to gains in prediction accuracy on the AUC scale of 9% (computed as 100 × [0.779/0.715–1]) for CD and 7.4% (computed as 100 × [0.741/0.690–1]) for UC when models were trained on iChip (Additional file 5: Table S2) and gains were slightly higher for gChip models. BayesR consistently outperformed the other methods in subsequent analyses and we therefore mainly report the BayesR results from hereon.

Prediction accuracy for CD and UC from 5-fold cross-validation was high, despite the low number of 42,534 markers (AUC 0.779 and 0.741 for CD and UC, respectively). This was expected as the SNP list contained GWAS hit SNPs. However, models trained on gChip had higher AUC for CD (0.806) and UC (0.766) than models based on iChip. If SNPs on both chips are without genotyping and imputation errors, accuracies are expected to be identical between chips. Lower accuracies for ichip could be partly due to missing genotypes, but the average missing rate of iChip SNPs was less than 0.05%. Another potential factor that could lead to systematic differences between iChip and gChip is confounding of case-control status by batch effects. Batch effects are possibly larger for gChip due to the use of different GWAS arrays and the splitting of cases and controls into 6 imputation cohorts for CD and 7 cohorts for UC. We looked for batch effects in the data by training models using gChip genotypes and then predicting the left out test set using the iChip genotypes and vice versa (Additional file 6: Figure S3). In the absence of systematic differences between genotypes, we would expect similar accuracies for the same validation sample irrespective which array was used for training. Using either gChip or iChip genotypes for validation did not change the predictive performance of models trained on iChip, whereas using gChip in both discovery and validation led to a gain in accuracy. For example for CD measured on the 0–1 scale, training on gChip or iChip and using iChip genotypes in the validation sample gave AUC of 0.771 and 0.779, respectively, compared to AUC of 0.806 and 0.776 when using gChip in the validation sample. This suggests a small artificial gain in the prediction performance for gChip in cross-validation, most likely due to imperfect imputation of SNPs not genotyped across all GWAS platforms.

To avoid prediction bias due to potential confounding effects from population stratification, we used adjusted phenotypes controlled for projected eigenvectors derived from the POPRES reference population ([24], Additional file 3: Figure S2). Using these adjusted phenotypes, it was observed that AUC values for CD and UC decreased by 3.2% (computed as 100 × [0.806/0.781–1]) and 6.8% (computed as 100 × [0.766/0.717–1]) for gChip and 3.3% (computed as 100 × [0.778/0.753–1]) and 6.8% (computed as 100 × [0.741/0.694–1]) for iChip, respectively.

### Prediction performance with increasing sample size and number of SNPs

**Fig. 3:**
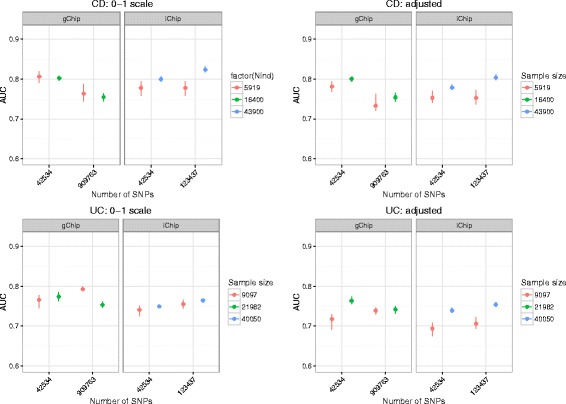
Prediction performance with increasing sample size and SNP density using BayesR. Prediction accuracy is measured as the area under the curve (AUC) with higher values denoting better performance. Prediction models were trained using either disease status (0–1) or disease phenotype adjusted for ancestry (adjusted)

We next investigated by 5-fold CV if increase in sample size and in the number of potential predictors makes a contribution to prediction performance (Fig. 3, Additional file 5: Table S2). A noticeable feature of Fig. 3 is that substantial increases in SNP density for both chips did not translate into big increases in AUC. The effective number of independent SNPs was estimated to be 2750 for the 42 K SNP set, 2986 for iChip and 37,226 for GWAS gChip. The increase in the effective number of independent SNPs of ~9% for iChip largely reflects the considerable LD between SNPs in regions of high density around the 163 susceptibility loci detected in the study described by Jostins et al. [2]. Surprisingly, the substantial increase in the number of independent SNPs for gChip had a negative effect on prediction performance in most scenarios for all methods (Additional file 5: Table S2). The SNPs in the 42 K set are very much enriched for specific regions in the genome known to be associated with IBD and this perhaps explains the observation for gChip, since the effects of all the extra SNPs with presumably zero, or very small contribution, have to be balanced with noise, potentially impacting negatively on prediction performance. In addition, we cannot rule out that other sources of artefactual confounding between discovery and target samples that escaped our QC contribute to the unexpected results for gChip.

### Prediction performance for the independent ANZ cohort

Within-study or cross-validation will most likely not reflect the exact performance of out-of-sample prediction. A proper assessment requires external validation in several datasets collected from different sources to avoid over-optimistic prediction results. After the previous GWAS [10, 11, 25–27], genotyping in IIBDGC was almost exclusively done with iChip and hence no independent dataset was available to assess the performance based on gChip samples. To evaluate models based on iChip we used the ANZ cohort as an independent validation population. The ANZ cohort includes samples recruited from centers within Australia and New Zealand that were excluded from the aforementioned cross-validation analyses. Further, the ANZ cohort was not included in the previous GWAS studies, which is important to protect against potential bias that would result from including individuals in the validation set that were also part of the data used for selecting SNPs onto iChip.

We used each of the five prediction models from 5-fold cross-validation to predict case-control status and we reported the mean AUC. In contrast to the results from cross-validation, models trained with adjusted phenotypes controlled for population stratification had similar performance to those with unadjusted phenotypes (Additional file 7: Table S3). This shows that adjustment for population structure is important to reduce the inflation of prediction accuracy when future accuracy is assessed by cross-validation.

Of the four methods, BayesR performed best across training sets varying in sample size and number of SNPs. Across the eight different data schemes, BayesR gave the highest AUC 5 times, GBLUP twice, and EN once (Additional file 8: Table S4). Overall, the gain in prediction performance by increasing sample size and number of SNPs was larger than what would be expected from the cross-validation results. Prediction models that were trained using all available individuals and SNPs had the best performance with AUC scores of 0.78 for CD and of 0.70 for UC, respectively. The prediction accuracy was lowest for models derived from the 42 K SNPs set and the smallest sample size (5919 CD samples, 9097 UC samples). Relative to this model, using all iChip markers (123,437 SNP) and increasing sample size to 43,900 for CD and 40,050 for UC led to gains in accuracy on the AUC scale of 9.9% for CD (computed as 100 × [0.746/0.679–1]) and 9.3% for UC (computed as 100 × [0.696/0.637–1]), respectively.

GPRS models included all available SNPs and had poor prediction performance. We investigated if performance using iChip would benefit from selection of markers at various *P*-value cutoffs (Additional file 9: Figure S4). For most CD training datasets, the maximum AUC was obtained for models including all SNPs, or improved only slightly using selection cutoffs. A similar trend for CD was reported for GPRS constructed from GWAS SNPs using the Wellcome Trust Case Control Consortium dataset [5]. AUC for UC improved by less than 0.022 for two of the four training sets when SNP were selected on *P*-value.

**Fig. 4:**
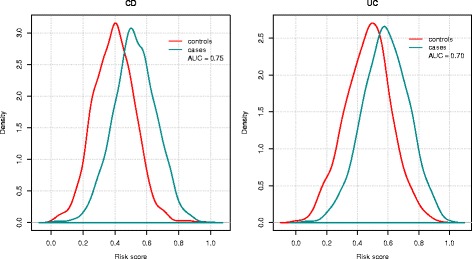
Distribution of genomic risk scores in UC and CD cases and controls of ANZ cohort. Kernel density estimates of risks scores in case and control groups predicted using models trained on IIBDGC samples and iChip

In Fig. 4 we plotted the kernel density estimates of the predicted risk scores for control and case groups based on the best performing model for BayesR. There was substantial separation of the cases from the controls for both diseases. As expected from the lower AUC, the separation was less profound for UC.

### Association of genomic risk score with clinical characteristics of Crohn’s disease

For 823 CD cases in the ANZ cohort additional clinical characteristics were available (Additional file 4: Table S1). We found that individuals with higher genomic risk scores more often required bowel resection (*P*-value <0.03), were younger at disease onset (*P*-value <0.005), and suffered more often from ileal than from colonic CD (*P*-value <0.003, Additional file 10: Figure S5). The *P-*value for disease onset and disease location is significant after Bonferoni adjustment for the three features investigated.

### Clinical applications of genomic risk score in the ANZ cohort

**Fig. 5:**
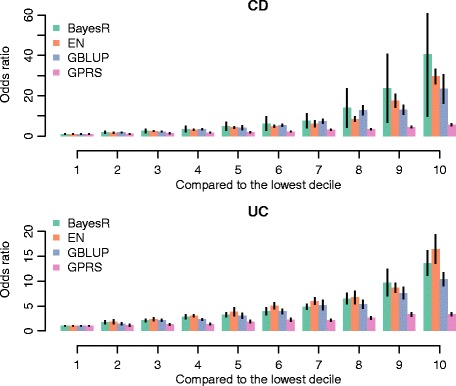
Odds ratio of case-control status. Individuals in the independent ANZ cohort were partitioned into 10 groups on the basis of the rank of their predicted risk score from BayesR, EN, GBLUP, and GPRS. The first decile is used as the reference group. The vertical bars denote mean and 95% confidence intervals from 5-fold cross-validation. The discovery populations included 123,437 iChip SNPs and 43,900 and 40,050 individuals for CD and UC, respectively

To quantify the usefulness of the genomic risk score, we compared the top and bottom 10% of the genetic risk predictors in the ANZ cohort using an epidemiological approach [16, 28]. Individual genetic risk scores were ranked from lowest to highest, and stratified into deciles. We obtained the odds ratio of case-control status for each decile comparing it to the lowest decile as a reference (Fig. 5). As expected, the odds ratio was largest for the difference between the 1st and the 10th decile. The odds ratio for CD between highest and lowest decile was 40.64 ± 31 for BayesR, 29.56 ± 3.62 for EN, 23.43 ± 7.09 for GBLUP and 5.69 ± 0.47 for GPRS, respectively. These observed values agree reasonably well with the expected odds ratio of 31.97, 26.75, and 7.4 given the observed AUC and assuming a prevalence of 0.005 for CD [29]. A value of 40 means that if a person’s risk profile score falls into the last decile he/she is 40 times more likely to be a case than if he/she belonged to the first decile. Utility of genomic risk scores for UC was lower with odds ratios of 13.62 ± 2.42, 16.45 ± 2.84, 10.39 ± 1.32 and 3.35 ± 0.15 for BayesR, EN, GBLUP and GPRS, respectively (Fig. 5). Assuming a prevalence of 0.002 for UC, these values again agree well with the expected odds ratio between highest and lowest decile of 13.69, 14.56, 11.93, and 4.44, respectively.

## Discussion

In this study we show that genomic risk scores estimated from a large discovery population can increase the prediction accuracy of an individual’s risk of CD and UC. It is not possible to compare all rivaling methods available for SNP-based prediction. We chose methods that were expected to run efficiently on the largest datasets of this study. Across various training sets the BayesR method outperformed other methods (GBLUP, EN, GPRS) in comparison. The good performance of BayesR is consistent with recent analyses that demonstrated that using a mixture of distributions for the SNP effects increases prediction accuracy for diseases with strong associations [20–22, 30].

Elastic net regularisation (EN) performed relatively poorly. In a recent study, based largely on the same iChip samples and using EN, Wei et al. [8] reported AUCs of 0.86 for CD and 0.83 for UC. Our reported CV estimate of 0.83 for CD is slightly lower; however for UC the best AUC we achieved was 0.77. A direct comparison of both studies is difficult since there were differences in QC protocols, the composition of discovery and validation samples and importantly, the SNPs available on iChip. We were not able to test the prediction model in Wei et al. [8], which included 573 and 366 single SNPs for CD and UC, respectively, as our validation samples (ANZ cohort) were included in their study. In addition, the iChip data we downloaded (iChip release 5, November, 2012) included 2113 fewer SNPs than the set that passed QC in Wei et al., and only 75% of the CD predictor SNPs and 73% of the UC predictor SNPs provided by Wei et al. [8]. Of the remaining predictors a further 11% for CD and 12% for UC failed our stringent QC. However, even with a substantial proportion of SNPs missing, one would still expect that a significant part of the original signals be tagged by other SNPs in LD, particularly in the high-density regions.

We used AUC to summarize the prediction performance across methods. However, in practice a prediction model should also be calibrated, that is, return a risk score that is on the same scale as the actual observed phenotype. For example, if a model predicts risk scores in the range from −0.5 to 0.5, but phenotypes are coded as 0 and 1, then the model is not calibrated, regardless how high AUC may be. BayesR and GBLUP risk scores are reasonably well calibrated whereas EN and GPRS scores are not. Calibration is important when genomic predictions are to be combined with other information sources.

Comparing prediction performance of the selected 42,534 SNPs subset with the full iChip (123,437 SNPS) and gChip (909,763 SNPs) set from cross-validation demonstrated that the power of iChip is mainly due to the power of the initial GWAS study for its marker selection. This suggests that residual associated SNPs that are only tagged by gChip and that low or rare-variants overrepresented in the high-density region on iChip contribute little to prediction accuracy.

About 25% of the SNP-heritability tagged by gChip SNPs is lost using iChip [12], but the decrease of the proportion of variance explained did not translate into decreased prediction performance. This observation is consistent with theoretical and empirical studies [20, 22, 31–33] that show prediction performance can be markedly different from the proportion of variance accounted for in the training set, particularly for traits with strong SNP associations. In the external validation using data from the ANZ cohort, increasing sample size resulted in gains in accuracy of 8% for CD and 9% for UC, largely consistent with the expectation that iChip increases power by enabling larger sample sizes. The highly shared etiology between CD and UC [2] could allow combining CD and UC cases into a much larger training dataset that is expected to further increase the power of risk stratification for IBD.

We have identified several potential sources of confounding like batch effects and divergent ancestry of individuals and show that they contribute very little to the prediction performance of the genomic risk predictor in the independent ANZ cohort. Although accuracies in the ANZ cohort were lower than those from cross-validation, we demonstrated the ability of the genomic risk score to discriminate between clinically relevant low-risk and high-risk groups. Even small increases in predictive ability can substantially increase the odds ratio of disease status for patients with the highest and lowest prediction scores.

CD and UC are heterogeneous complex phenotypes in terms of age of onset, disease location and disease behavior [34, 35]. Disease heterogeneity poses a challenge in developing accurate genomic risk predictors from case-control studies [36]. To develop a risk score that is predictive in all patients, larger sample sizes are needed to ensure that relevant subtypes have adequate representation in the case-control study. One potential solution is to conduct ‘deep phenotyping’, but this might not be achievable in retrospect. A more realistic option is to collect detailed phenotypes on new cases and to then stratify samples based on their genetic risk score [37]. For example, we found that higher genomic risk scores for CD were associated with clinical characteristics typically associated with increased disease severity, including ileal location and younger age of onset [38, 39]. Although this approach did not achieve the separation required for diagnostic purposes, it could be used to stratify cases into relevant subgroups at diagnosis for further prospective, longitudinal studies to identify additional factors that determine a severe disease course [40].

Our analysis of deciles in the ANZ cohort confirms that there may also be clinical utility in using genetic risk scores at the extremes, specifically at the higher end of the scale [39]. Currently there are no clinical guidelines for screening unaffected first-degree relatives of patients with either CD or UC, unlike those set out for colorectal cancer. First-degree relatives of patients with either IBD and with a high genetic risk score may be considered for simple, non-invasive, and inexpensive screening tests such as an annual or biannual fecal calprotectin [41]. Prospective studies will be needed to determine the utility and cost-effectiveness of such a strategy as compared to current established strategies such as those for first-degree relatives of patients with colorectal cancer.

## Conclusions

Implementing genomic risk prediction for IBD in clinical practise involves making important decisions regarding the choice of model, the size of the training data and the SNP genotyping array. We demonstrate benefits in prediction performance using a Bayesian mixture model that takes advantage of the known genetic architecture for CD and UC. Our analyses demonstrate that the power of genomic risk prediction for CD and UC is mainly due to strongly associated SNPs with considerable effect sizes. Additional SNPs only tagged by high-density GWAS arrays and low or rare-variants over-represented in the high-density region on the Immunochip contribute little to prediction accuracy. These results favour the Immunochip over GWAS chips as it facilitates larger sample sizes.

Individualised risk assessment is an important concept in an era of personalised medicine. In clinical practise, the genomic risk score has little utility to diagnose IBD in individuals, largely because the diseases are highly polygenic. Rather, genomic risk scores provide additional risk stratification that is not fully captured with currently available clinical information at diagnosis. By identifying individuals with heightened genetic risk clinicians can recommend earlier and more frequent clinical assessment allowing more effective interventions or treatment options both in patients and in other high risk groups such as first-degree relatives of those with IBD.

## Additional files

Additional file 1: Members of the International IBD Genetics Consortium. (XLS 48 kb)

Additional file 2: Figure S1. Prediction accuracy (AUC) of GBLUP and multiBLUP for CD and UC (0–1 scale) from cross-validation depending on genotyping chip, sample size and number of SNPs. (TIFF 68 kb)

Additional file 3: Figure S2. Principal Component analysis for CD and UC. We obtained the first ten principal components from a reference sample of 2466 self-reported Europeans downloaded from the POPRES collection using 608,435 SNPs. The inferred ancestry of the samples agreed well with country of origin of the samples and therefore we reason that sample quality control was sufficient. a. The projected PC for CD gChip samples recruited from Belgium, The Children’s Hospitcal of Phildelphia (USA), Germany, National Institute of Diabetes and Digestive and Kidney Diseases (NIDDK, USA), and WTCCC (UK). b. The projected PC for UC gChip samples from the Children’s Hospitcal of Phildelphia (USA), Germany, Norway, Sweden, and WTCCC (UK). c. The principal component coordinates for POPRES samples from countries similar to the IBD samples. (TIFF 3988 kb)

Additional file 4: Table S1. ANZ CD cases with additional clinical characteristics. (DOCX 13 kb)

Additional file 5: Table S2. Prediction accuracy (AUC) for CD and UC (0–1 scale) from cross-validation depending on prediction method, genotyping chip, sample size and number of SNPs. (DOCX 18 kb)

Additional file 6: Figure S3. Batch effect analysis for CD and UC. We used samples genotyped with both iChip and gChip and extracted 42,534 SNPs in common between both platforms. We looked for batch effects in the data by training a model using gChip and then predicting the left out test set using the iChip genotypes and vice versa. In the absence of systematic differences we would expect the same accuracies for the same test set regardless if the model was trained on iChip or gChip. As shown, using gChip for discovery and validation gave higher accuracies, indicating that even after stringent QC performance estimates are still biased by batch effect confounding. (TIFF 673 kb)

Additional file 7: Table S3. Prediction accuracy (AUC) of BayesR for CD and UC in ANZ cohort depending on sample size, number of iChip SNPs and phenotype. (DOCX 15 kb)

Additional file 8: Table S4. Prediction accuracy (AUC) for CD and UC in ANZ cohort depending on prediction method, sample size and number of iChip SNPs. (DOCX 15 kb)

Additional file 9: Figure S4. Effect of *P*-value cutoff on prediction performance of GPRS. (TIFF 56 kb)

Additional file 10: Figure S5. Distribution of genomic risk scores for CD in groups stratified for severity of disease. Kernel density estimates of normalized risks scores in 823 CD cases of the ANZ cohort predicted using models trained on case-control status using IBDGC samples and iChip SNPS. (TIFF 81 kb)

Additional file 11: List of Ethics Approvals. (DOC 33 kb)

## Abbreviations

ANZ cohort: Independent validation cohort of Australian and New Zealand individuals
AUC: Area under the curve
BayesR: Bayesian hierarchical method that models SNP effects as a mixture of normal distributions
CD: Crohn’s disease
CV: Cross-validation
EN: Elastic net
GBLUP: Genomic best linear unbiased prediction
gChip: GWAS SNP chip
GPRS: Genetic profile risk score
GWAS: Genome-wide association study
IBD: Inflammatory bowel disease
iChip: Immunochip
IIBDGC: The International Inflammatory Bowel Disease Genetics Consortium
INFO score R^2^: Information metric indicating the imputation quality of a SNP
LD: Linkage disequilibrium
MAF: Minor allele frequency
MCMC: Markov Chain Monte-Carlo
MTG2: Software for multivariate linear mixed model analysis using genomic information, including GBLUP
multiBLUP: Extends GBLUP to include multiple random effects
POPRES: The population reference sample
QC: Quality control
ROC curve: Receiver-operating characteristic curve
SNP: Single nucleotide polymorphism
UC: Ulcerative colitis
