# Supplementary figures and images for: Performance of risk prediction for inflammatory bowel disease based on genotyping platform and genomic risk score method
Source: BMC Med Genet. 2017 Aug 29;18:94. doi: 10.1186/s12881-017-0451-2 (PMC5576242; doi:10.1186/s12881-017-0451-2)

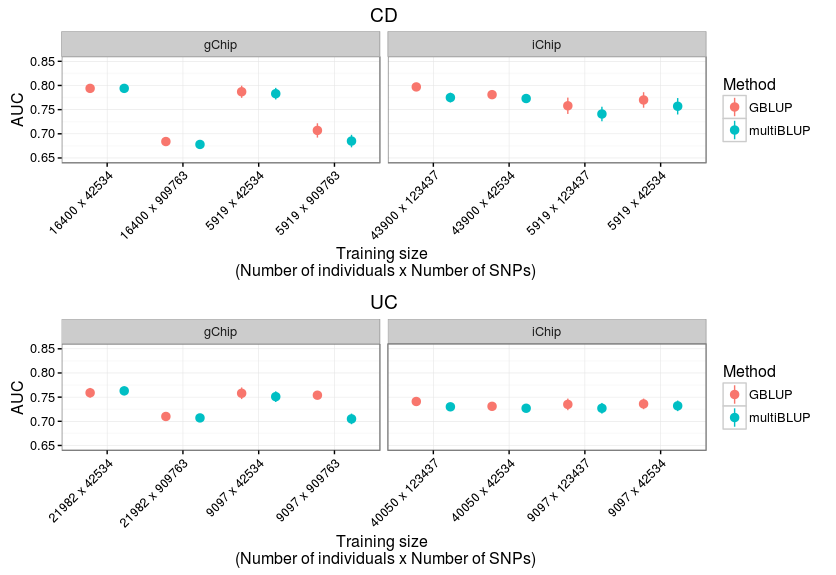

Supplement: Supplementary file 2 — Prediction accuracy (AUC) of GBLUP and multiBLUP for CD and UC (0–1 scale) from cross-validation depending on genotyping chip, sample size and number of SNPs. (TIFF 68 kb) [file 12881_2017_451_MOESM2_ESM.tif]

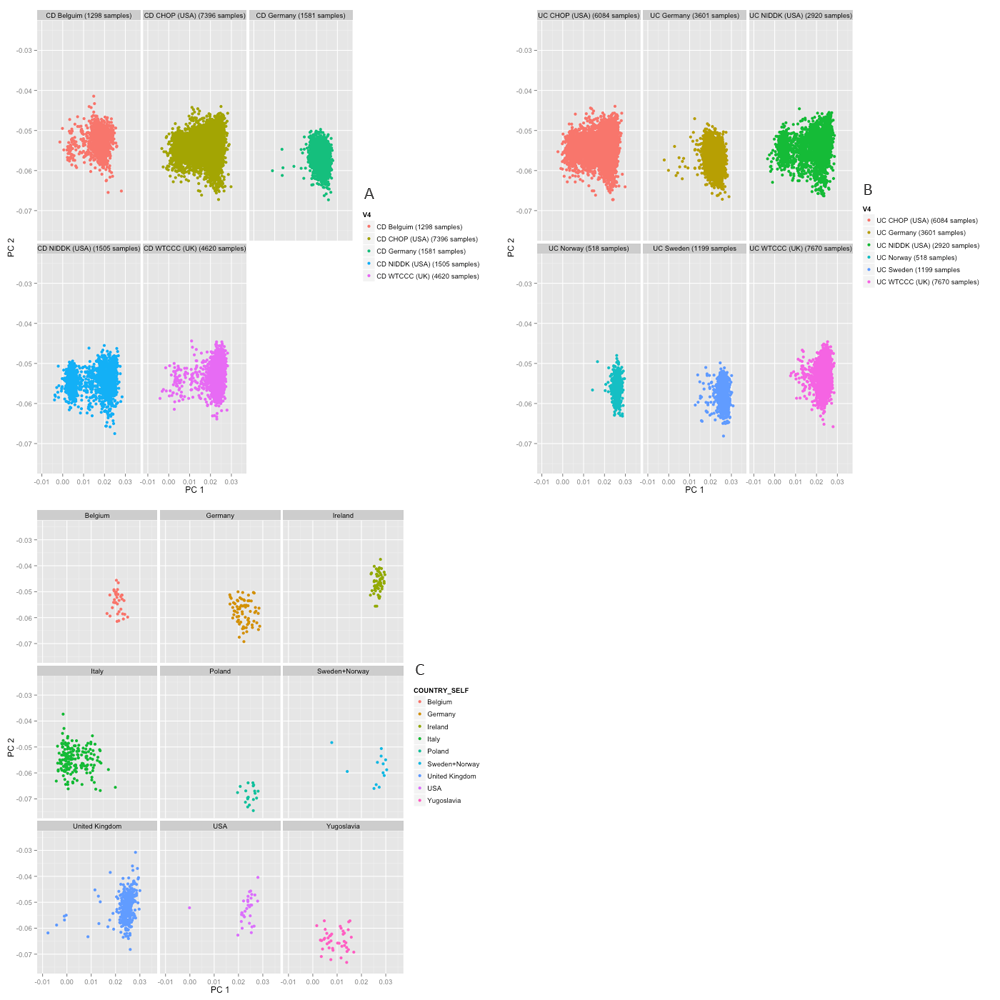

Supplement: Supplementary file 3 — Principal Component analysis for CD and UC. We obtained the first ten principal components from a reference sample of 2466 self-reported Europeans downloaded from the POPRES collection using 608,435 SNPs. The inferred ancestry of the samples agreed well with country of origin of the samples and therefore we reason that sample quality control was sufficient. a. The projected PC for CD gChip samples recruited from Belgium, The Children’s Hospitcal of Phildelphia (USA), Germany, National Institute of Diabetes and Digestive and Kidney Diseases (NIDDK, USA), and WTCCC (UK). b. The projected PC for UC gChip samples from the Children’s Hospitcal of Phildelphia (USA), Germany, Norway, Sweden, and WTCCC (UK). c. The principal component coordinates for POPRES samples from countries similar to the IBD samples. (TIFF 3988 kb) [file 12881_2017_451_MOESM3_ESM.tif]

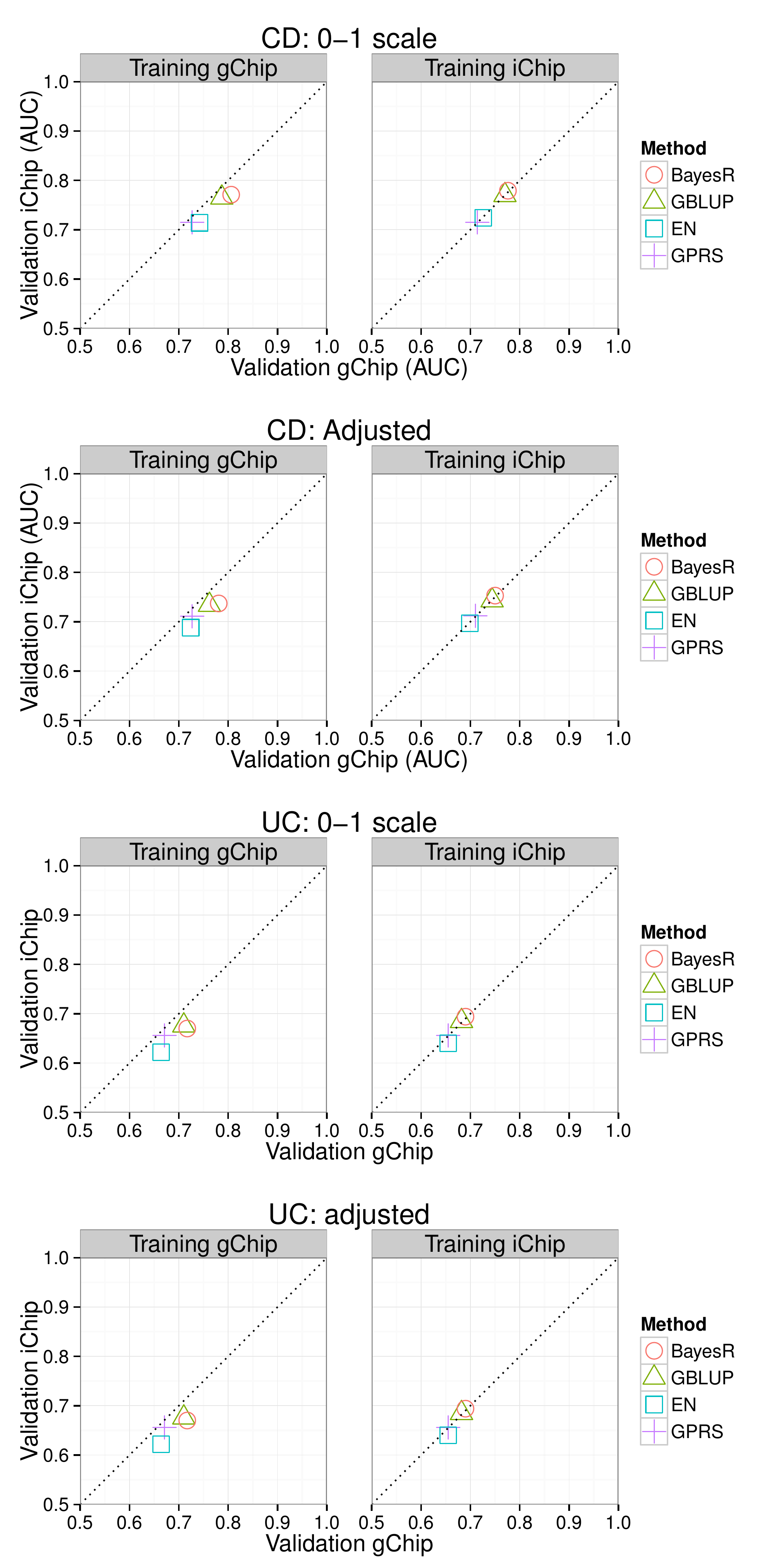

Supplement: Supplementary file 6 — Batch effect analysis for CD and UC. We used samples genotyped with both iChip and gChip and extracted 42,534 SNPs in common between both platforms. We looked for batch effects in the data by training a model using gChip and then predicting the left out test set using the iChip genotypes and vice versa. In the absence of systematic differences we would expect the same accuracies for the same test set regardless if the model was trained on iChip or gChip. As shown, using gChip for discovery and validation gave higher accuracies, indicating that even after stringent QC performance estimates are still biased by batch effect confounding. (TIFF 673 kb) [file 12881_2017_451_MOESM6_ESM.tif]

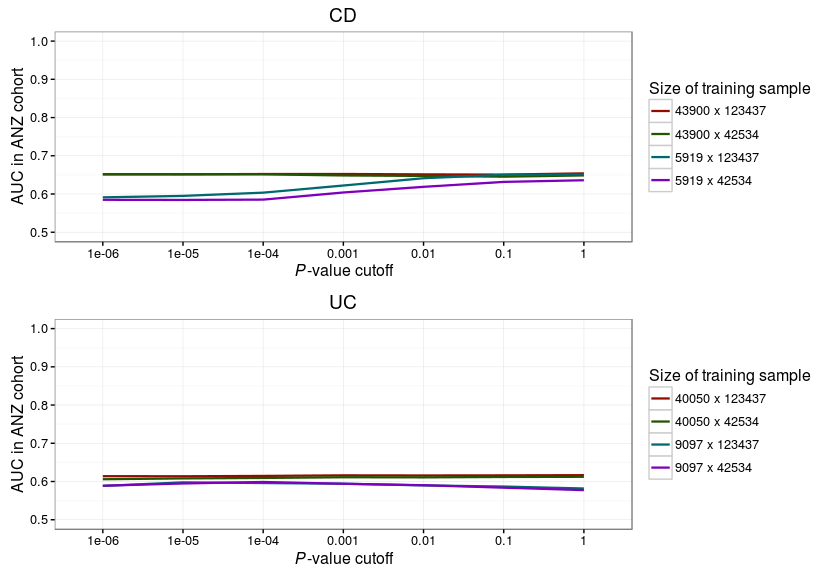

Supplement: Supplementary file 9 — Effect of P-value cutoff on prediction performance of GPRS. (TIFF 56 kb) [file 12881_2017_451_MOESM9_ESM.tif]

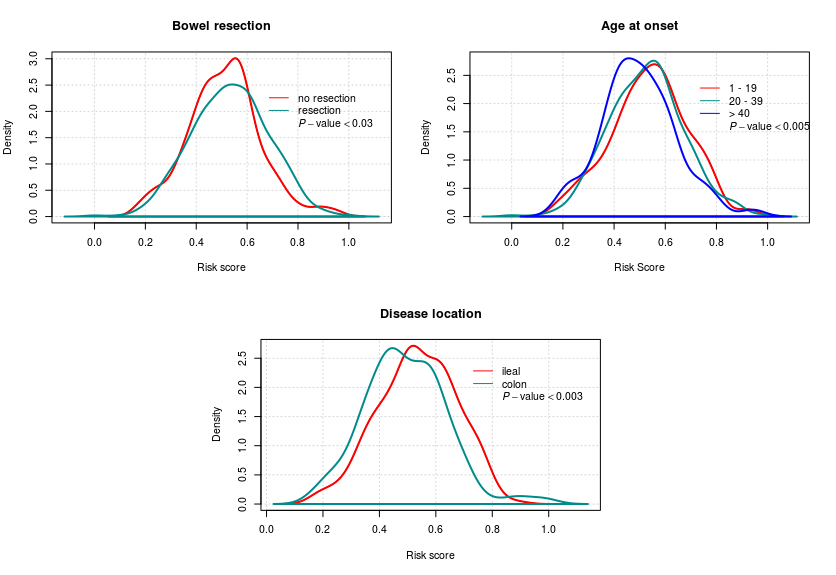

Supplement: Supplementary file 10 — Distribution of genomic risk scores for CD in groups stratified for severity of disease. Kernel density estimates of normalized risks scores in 823 CD cases of the ANZ cohort predicted using models trained on case-control status using IBDGC samples and iChip SNPS. (TIFF 81 kb) [file 12881_2017_451_MOESM10_ESM.tif]
