# Supplementary material for: Performance of risk prediction for inflammatory bowel disease based on genotyping platform and genomic risk score method
Source: BMC Med Genet. 2017 Aug 29;18:94. doi: 10.1186/s12881-017-0451-2 (PMC5576242; doi:10.1186/s12881-017-0451-2)
Supplement: Supplementary file 4 — ANZ CD cases with additional clinical characteristics. (DOCX 13 kb) [file 12881_2017_451_MOESM4_ESM.docx]

**Table S1. ANZ CD cases with additional clinical characteristics**

| **CROHN'S DISEASE (n=823)** | | |
| --- | --- | --- |
| **Age at diagnosis** | Mean (years)  1 – 19  20 – 39  40+ | 27.74  215 (26.1%)  478 (58.1%)  130 (15.8%) |
| **Disease Location (cumulative)** | Ileal (L1) | 240 (29.2%) |
|  | Colorectal (L2) | 159 (19.3%) |
|  | Ileocolonic (L3) | 424 (51.5%) |
| **Bowel Resection** | No | 329 (40.0%) |
|  | Yes | 494 (60.0%) |
