# Supplementary material for: Performance of risk prediction for inflammatory bowel disease based on genotyping platform and genomic risk score method
Source: BMC Med Genet. 2017 Aug 29;18:94. doi: 10.1186/s12881-017-0451-2 (PMC5576242; doi:10.1186/s12881-017-0451-2)
Supplement: Supplementary file 5 — Prediction accuracy (AUC) for CD and UC (0–1 scale) from cross-validation depending on prediction method, genotyping chip, sample size and number of SNPs. (DOCX 18 kb) [file 12881_2017_451_MOESM5_ESM.docx]

**Table S2.** Prediction accuracy (AUC) for CD and UC (0-1 scale) from cross-validation depending on prediction method, genotyping chip, sample size and number of SNPs

| Disease | Samples | SNPs | Chip | GPRS | GBLUP | EN | BayesR |
| --- | --- | --- | --- | --- | --- | --- | --- |
| CD | 5,919 | 42,534 | iChip | 0.715 | 0.770 | 0.724 | **0.779** |
|  |  |  |  | (0.0156) | (0.0161) | (0.0144) | (0.0147) |
|  | 5,919 | 42,534 | gChip | 0.727 | 0.787 | 0.742 | **0.806** |
|  |  |  |  | (0.0140) | (0.0126) | (0.0100) | (0.0130) |
|  | 5,919 | 123,437 | iChip | 0.722 | 0.758 | 0.723 | **0.777** |
|  |  |  |  | (0.0158) | (0.0169) | (0.0139) | (0.0159) |
|  | 5,919 | 909,763 | gChip | 0.694 | 0.707 | 0.699 | **0.763** |
|  |  |  |  | (0.0117) | (0.0148) | (0.0148) | (0.0180) |
|  | 16,400 | 42,534 | gChip | 0.719 | 0.794 | 0.771 | **0.803** |
|  |  |  |  | (0.0087) | (0.0027) | (0.0029) | (0.0046) |
|  | 16,400 | 909,763 | gChip | 0.676 | 0.684 | 0.683 | **0.755** |
|  |  |  |  | (0.0134) | (0.0057) | (0.0156) | (0.0089) |
|  | 43,900 | 42,534 | iChip | 0.714 | 0.781 | 0.775 | **0.799** |
|  |  |  |  | (0.0159) | (0.0065) | (0.0039) | (0.0047) |
|  | 43,900 | 123,437 | iChip | 0.700 | 0.797 | 0.781 | **0.825** |
|  |  |  |  | (0.0068) | (0.0058) | (0.0063) | (0.0036) |
|  |  |  |  |  |  |  |  |
| UC | 9,097 | 42,534 | iChip | 0.690 | 0.736 | 0.690 | **0.741** |
|  |  |  |  | (0.0120) | (0.0114) | (0.0114) | (0.0102) |
|  | 9,097 | 42,534 | gChip | 0.698 | 0.758 | 0.709 | **0.766** |
|  |  |  |  | (0.0123) | (0.0115) | (0.0099) | (0.0124) |
|  | 9,097 | 123,437 | iChip | 0.694 | 0.735 | 0.713 | **0.755** |
|  |  |  |  | (0.0076) | (0.0122) | (0.0105) | (0.0108) |
|  | 9,097 | 909,763 | gChip | 0.716 | 0.754 | 0.734 | **0.792** |
|  |  |  |  | (0.0059) | (0.0035) | (0.0134) | (0.0048) |
|  | 21,982 | 42,534 | gChip | 0.675 | 0.759 | 0.741 | **0.774** |
|  |  |  |  | (0.0109) | (0.0104) | (0.0103 | (0.0093) |
|  | 21,982 | 909,763 | gChip | 0.676 | 0.710 | 0.712 | **0.753** |
|  |  |  |  | (0.0081) | (0.0079) | 0.0022) | (0.0056) |
|  | 40,050 | 42,534 | iChip | 0.648 | 0.731 | 0.724 | **0.749** |
|  |  |  |  | (0.0065) | (0.0027) | (0.0054) | (0.0041) |
|  | 40,050 | 123,437 | iChip | 0.673 | 0.741 | 0.734 | **0.764** |
|  |  |  |  | (0.0133) | (0.0038) | (0.0045) | (0.0042) |

The AUC of the best performing method for each scenario is highlighted in bold. Values are means and standard deviations (parenthesis) from 5-fold cross-validation.
