# Supplementary material for: Performance of risk prediction for inflammatory bowel disease based on genotyping platform and genomic risk score method
Source: BMC Med Genet. 2017 Aug 29;18:94. doi: 10.1186/s12881-017-0451-2 (PMC5576242; doi:10.1186/s12881-017-0451-2)
Supplement: Supplementary file 7 — Prediction accuracy (AUC) of BayesR for CD and UC in ANZ cohort depending on sample size, number of iChip SNPs and phenotype. (DOCX 15 kb) [file 12881_2017_451_MOESM7_ESM.docx]

**Table S3.** Prediction accuracy (AUC) of BayesR for CD and UC in ANZ cohort depending on sample size, number of iChip SNPs and phenotype

|  |  |  | Phenotype | |
| --- | --- | --- | --- | --- |
| Disease | Samples | SNPs | 0-1 scale | Adjusted |
| CD | 5,919 | 42,534 | 0.679 (0.0043) | 0.671  (0.0047) |
|  |  |  |  |  |
|  | 43,900 | 42,534 | 0.700 (0.0112) | 0.707  (0.0156) |
|  |  |  |  |  |
|  | 5,919 | 123,437 | 0.696 (0.0039) | 0.686  (0.0044) |
|  |  |  |  |  |
|  | 43,900 | 123,437 | 0.746 (0.0411) | 0.703  (0.0320) |
|  |  |  |  |  |
| UC | 9,097 | 42,534 | 0.637 (0.0060) | 0.656  (0.0054) |
|  |  |  |  |  |
|  | 40,050 | 42,534 | 0.648 (0.0089) | 0.638  (0.0118) |
|  |  |  |  |  |
|  | 9,097 | 123,437 | 0.658 (0.0106) | 0.656  (0.0337) |
|  |  |  |  |  |
|  | 40,050 | 123,437 | 0.696 (0.0146) | 0.692  (0.0167) |

Values are means and standard deviations (parenthesis) from 5-fold cross-validation. Phenotypes were adjusted for the top 10 principal components (see Figure S1 for details).
