# Supplementary material for: Performance of risk prediction for inflammatory bowel disease based on genotyping platform and genomic risk score method
Source: BMC Med Genet. 2017 Aug 29;18:94. doi: 10.1186/s12881-017-0451-2 (PMC5576242; doi:10.1186/s12881-017-0451-2)
Supplement: Supplementary file 8 — Prediction accuracy (AUC) for CD and UC in ANZ cohort depending on prediction method, sample size and number of iChip SNPs. (DOCX 15 kb) [file 12881_2017_451_MOESM8_ESM.docx]

**Table S4.** Prediction accuracy (AUC) for CD and UC in ANZ cohort depending on prediction method, sample size and number of iChip SNPs

| Disease | Samples | SNPs | GPRS | GBLUP | EN | BayesR |
| --- | --- | --- | --- | --- | --- | --- |
| CD | 5,919 | 42,534 | 0.636 (0.0051) | 0.665  (0.0038) | 0.620 (0.0062) | **0.679 (0.0043**) |
|  |  |  |  |  |  |  |
|  | 43,900 | 42,534 | 0.648 (0.0020) | **0.712 (0.0045)** | 0.703 (0.0060) | 0.700 (0.0112) |
|  |  |  |  |  |  |  |
|  | 5,919 | 123,437 | 0.651 (0.0019) | 0.672 (0.0042) | 0.643 (0.0107) | **0.696 (0.0039)** |
|  |  |  |  |  |  |  |
|  | 43,900 | 123,437 | 0.654 (0.0022) | 0.736 (0.0039) | 0.736 (0.0060) | **0.746 (0.0411)** |
|  |  |  |  |  |  |  |
|  |  |  |  |  |  |  |
| UC | 9,097 | 42,534 | 0.577 (0.0041) | 0.621 (0.0048) | 0.577 (0.0103) | **0.637 (0.0060)** |
|  |  |  |  |  |  |  |
|  | 40,050 | 42,534 | 0.612 (0.0028) | **0.670 (0.0044)** | 0.661 (0.0083) | 0.648 (0.0089) |
|  |  |  |  |  |  |  |
|  | 9,097 | 123,437 | 0.582 (0.0052) | 0.634 (0.0045) | 0.581 (0.0132) | **0.658 (0.0106)** |
|  |  |  |  |  |  |  |
|  | 40,050 | 123,437 | 0.617 (0.0025) | 0.687 (0.0040) | **0.700 (0.0045)** | 0.696 (0.0146) |

Values are means and standard deviations (parenthesis) from 5-fold cross-validation. The AUC of the best performing method for each scenario is highlighted in bold. Predictions are estimated from non-adjusted training models.
