# Supplementary material for: Performance of risk prediction for inflammatory bowel disease based on genotyping platform and genomic risk score method
Source: BMC Med Genet. 2017 Aug 29;18:94. doi: 10.1186/s12881-017-0451-2 (PMC5576242; doi:10.1186/s12881-017-0451-2)
Supplement: Supplementary file 11 — List of Ethics Approvals. (DOC 33 kb) [file 12881_2017_451_MOESM11_ESM.doc]

| **Centre** | **Location** | **Principal Investigator** | **Human Research Ethics Committee** | **Ethics approval number** |
| --- | --- | --- | --- | --- |
| Royal Brisbane & Women’s Hospital  QIMR Berghofer Medical Research Institute | Brisbane, QLD | Graham Radford-Smith | Royal Brisbane & Women’s Hospital Human Research Ethics Committee    QIMR Berghofer Human Research Ethics Committee | HREC/14/QRBW/323        H0311-080(P692) |
| Townsville Hospital | Townsville, QLD | Gillian Mahy | Townsville Hospital and Health Service Human Research Ethics Committee | HREC/09/QTHS/7 |
| Royal Adelaide Hospital | Adelaide, SA | Jane Andrews | Royal Adelaide Hospital Research Ethics Committee | 090311 |
| Flinders Medical Centre | Adelaide, SA | Peter Bampton | Southern Adelaide Clinical Human Research Ethics Committee | 13/08 |
| Fremantle Hospital/  St John of God Hospital | Perth, WA | Ian Lawrance | South Metropolitan Area Health Service Human Research Ethics Committee | 07/589 |
| Christchurch Hospital | Christchurch, NZ | Richard Gearry | Health and Disability Ethics Committees - Upper South A Regional Ethics Committee | CTY/03/01/011 |
| St Vincent’s Hospital | Sydney, NSW | Alissa Walsh | St Vincent’s Hospital Human Research Ethics Committee | HREC/12/SVH195 |
| Liverpool Hospital | Sydney, NSW | Susan Connor | South Western Sydney Local Health District Human Research Ethics Committee | HREC approval - HREC/12/SVH195 (approved under St Vincent's Hospital HREC)    Site specific approval - 13/018 |
| St Vincent’s  Hospital Melbourne | Melbourne, VIC | Sally Bell | St Vincent’s Hospital Melbourne Human Research Ethics Committee | HREC approval - HREC/12/SVH195 (approved under St Vincent's Hospital HREC)    Site specific approval - 019/13 |
| Alfred Hospital | Melbourne, VIC | Miles Sparrow | Alfred Hospital Ethics Committee | HREC approval - HREC/12/SVH195 (approved under St Vincent's Hospital HREC)    Site specific approval - 43/13 |
